# AnoPrimer: Primer Design in malaria vectors informed by range-wide genomic variation

**DOI:** 10.12688/wellcomeopenres.20998.1

**Published:** 2024-05-15

**Authors:** Sanjay C. Nagi, Faisal Ashraf, Alistair Miles, Martin J. Donnelly

**Affiliations:** 1 Department of Vector Biology, Liverpool School of Tropical Medicine, Liverpool, L3 5QA, UK; 2 Wellcome Sanger Institute, Hinxton, England, UK

**Keywords:** PCR, Anopheles, Primers, Probes, Molecular biology

## Abstract

The major malaria mosquitoes, *Anopheles gambiae s.l* and *Anopheles funestus*, are some of the most studied organisms in medical research and also some of the most genetically diverse. When designing polymerase chain reaction (PCR) or hybridisation-based molecular assays, reliable primer and probe design is crucial. However, single nucleotide polymorphisms (SNPs) in primer binding sites can prevent primer binding, leading to null alleles, or bind suboptimally, leading to preferential amplification of specific alleles. Given the extreme genetic diversity of *Anopheles* mosquitoes, researchers need to consider this genetic variation when designing primers and probes to avoid amplification problems. In this note, we present a Python package, AnoPrimer, which exploits the Ag1000G and Af1000 datasets and allows users to rapidly design primers in *An. gambiae* or *An. funestus,* whilst summarising genetic variation in the primer binding sites and visualising the position of primer pairs. AnoPrimer allows the design of both genomic DNA and cDNA primers and hybridisation probes. By coupling this Python package with Google Colaboratory, AnoPrimer is an open and accessible platform for primer and probe design, hosted in the cloud for free. AnoPrimer is available here https://github.com/sanjaynagi/AnoPrimer and we hope it will be a useful resource for the community to design probe and primer sets that can be reliably deployed across the *An. gambiae* and *funestus* species ranges.

## Plain Language Summary

The majority of molecular biology applications require synthetic DNA sequences called primers, which bind to DNA and allow us to amplify specific stretches of DNA which we are interested in. Unfortunately, when mutations occur at primer binding sites, primers can fail to bind completely, or even worse, amplify differentially depending on the mutation present. Mutations can therefore bias molecular assays with often undetected effects. This is a particular problem in malaria mosquitoes, as they are some of the most genetically diverse species on earth. We present a user-friendly software tool, AnoPrimer, which allows users to design primers for molecular biology in malaria mosquitoes. AnoPrimer integrates high-quality whole-genome sequence data from the cloud, and creates clear, interactive visualisations, enabling users to avoid mutations that occur in wild malaria mosquitoes. By avoiding these mutations, we can ensure the design of reliable primers which result in robust molecular assays for research into malaria vectors.

## Introduction

The polymerase chain reaction (PCR) is ubiquitous in molecular biology, providing template sequence for a wide array of techniques, such as detecting the presence or absence of particular DNA sequences, quantifying the abundance of transcripts, or in Sanger and next-generation sequencing. Primers - short, single-strand DNA sequences which bind to the template and facilitate amplification - are crucial to effective PCR reactions and must be designed to be robust, reliable and consistent across experimental conditions.

Single nucleotide polymorphisms (SNPs) in primer binding sites can affect both the stability of the primer-template duplex, as well as the efficiency with which DNA polymerases can extend the primer ( Letowski *et al.*, 2004; Wu *et al.*, 2009). In some cases, this can completely prevent primer binding and amplification of the template DNA, often referred to as null alleles or allelic dropout ( Carlson *et al.*, 2006). On most genotyping platforms, these alleles are problematic and difficult to detect, as null allele heterozygotes will be indistinguishable from true homozygous individuals. Allelic dropout is known to cause problems in human genetic testing ( Silva *et al.*, 2017; Zajícková *et al.*, 2003). Null allele homozygotes could be suggested if a sample repeatedly fails to amplify, however, when performing PCR on pooled samples we would not observe this failure, and therefore can never know whether all samples amplified successfully. Ensuring genetic markers do not violate Hardy-Weinberg equilibrium (HWE) is one way to partially safeguard against this problem ( Chapuis & Estoup, 2007), however, this is not always performed in practice, and excluding such markers may lead to loss of information when HWE deviation has another cause.

Another problematic scenario occurs if primers do bind but with unequal efficiency against different genetic variants. In this case, any quantitative molecular assay, such as qPCR for gene expression, could be severely affected and lead to biases in the estimation of sequence abundance between genetic variants or strains ( Lefever *et al.*, 2013). A previous study found that single mismatches can introduce a range of impacts on Cq values, ranging from relatively minor (<1.5) to major (>7.0) ( Stadhouders *et al.*, 2010). The impact of a variant on primer binding depends on multiple factors but mismatches within the last 5 nucleotides at the 3’ end can disrupt the nearby polymerase active site, and so these mismatches tend to have a much greater impact ( Martins *et al.*, 2011; Stadhouders *et al.*, 2010). Primers should therefore be designed to avoid these sites or if unavoidable, to contain degenerate bases at the sites of SNPs, in order to maximise the robustness of molecular experiments ( Quinlan & Marth, 2007).

The *Anopheles gambiae* 1000 genomes project has revealed staggering amounts of genetic variation in the major malaria mosquito, *Anopheles gambiae s.l ( Miles *et al*., 2017)* with a segregating SNP in less than every 2 bases of the accessible genome ( Ag1000G, 2020). The *An. funestus* 1000 genomes project is also underway ( https://www.malariagen.net/project/anopheles-funestus-genomic-surveillance-project/). Despite this, the vast majority of existing primers designed to target the *An. gambiae s.l* and *An. funestus* genomes do not consider SNP variation. In the past, this was not straightforward, as it would require both handling large genomic datasets and matching designed primers to genomic positions. Thanks to recent advances in cloud computing and the malariagen_data API, we can now design primers in the cloud whilst checking for genetic variation in the *An. gambiae* and *funestus* 1000 genomes projects. In this note we present AnoPrimer, a Python package which is coupled with a Google Colaboratory notebook, allowing users to easily design primers and probes in the cloud whilst considering genetic variation in major *Anopheles* vectors.

## Methods

### Implementation

AnoPrimer is a two-phase process, first designing sets of primers and probes and secondly investigating SNP variation in the targeted sites. AnoPrimer uses Primer3 as the core primer design engine, in the form of Primer3-py. Primer3 is open-source and has become the *de-facto* standard for primer design for molecular biology. Primer3-py is a set of recently developed Python bindings for the Primer3 program ( Untergasser *et al.*, 2012), which can be run readily in a Google Colaboratory environment. To load genetic variation data from the *Anopheles* 1000 genomes project, we integrate the malariagen_data API, which allows rapid download and analysis of genomic data from the cloud. Integration of the PyData stack in malariagen_data allows users to perform rapid genomic analysis on large datasets where compute resources are modest, such as in Google Colaboratory notebooks. Google Colaboratory is a proprietary version of Jupyter Notebook and is provided for free alongside CPU and GPU access to anyone with a Google account.

### Operation

**Figure 1.**
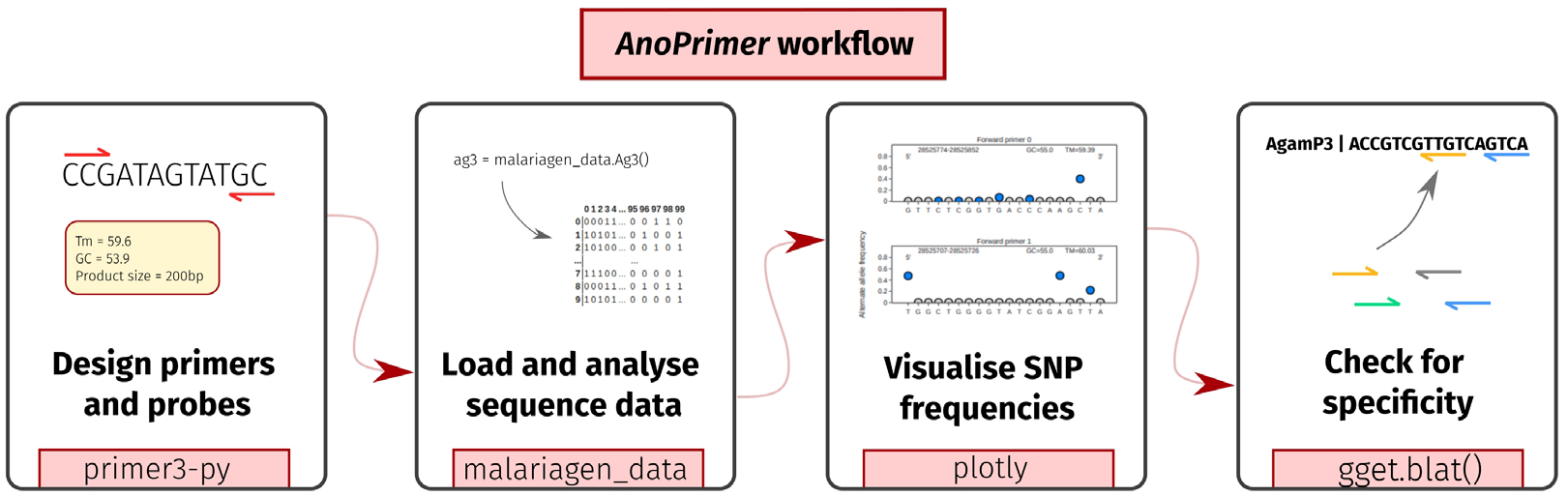
The AnoPrimer workflow. AnoPrimer first designs primers and probes with Primer3-py and then loads and visualises sequence data using the malariagen_data API, to allow users to decide on the most appropriate primer pairs. Finally, users can check for specificity by aligning their designed primers to the genome with the gget implementation of Blat.

***Overview of the workflow***. AnoPrimer can be run in two ways, either running the full Colaboratory notebook in a step-wise fashion, or in a single command which produces all outputs, which may be preferred in more high-throughput primer design settings. Users may select primer design parameters by providing a Python dictionary, or the primer3 default parameters can be used. Figure 1 shows the overall AnoPrimer workflow.

***Software requirements***. AnoPrimer is hosted on the Python package index (PyPi) and is compatible with Python versions 3.8, 3.9, 3.10, and 3.11, and with both Windows and Unix operating systems. We recommend that users have at least 4 GB of RAM and at least 1 GB of storage space. If AnoPrimer is used outside of Google Colaboratory, fast download speeds may be required to facilitate the rapid download of genomic data from the cloud. 

## Use cases

### Primer design with *primer3*

AnoPrimer allows the design of genomic DNA primers, hybridisation probes or cDNA primers (for gene expression purposes). In the case of cDNA primers, one of the forward or reverse primers will be designed to span an exon-exon junction where available, to prevent the amplification of genomic DNA in the sample.

Table 1 shows the output from the initial phase of primer design. AnoPrimer reports the primer sequences, along with information on melting point, GC content, amplicon size and position in the target sequence, though the full Primer3 output is accessible to the user. The user may specify the number of desired primer pairs to design. After the Primer3 run, AnoPrimer will print out run statistics which may be useful for troubleshooting.

**Table 1.** Primer3 results: A pandas dataframe and excel spreadsheet generated by AnoPrimer. Useful information from each primer set is stored, such as the sequence, melting temperature and GC content.

Primer Pair	0	1	2
**Primer forward sequence**	GTTCTCGGTGACCCAAGCTA	TGGCTGGGGTATCGGAGTTA	GGTGACCCAAGCTATACTGCA
**Primer reverse sequence**	GCGCTAGGGGTTGATCTCTC	TGCAGTATAGCTTGGGTCACC	ATTGGCGCTAGGGGTTGATC
**Primer forward TM**	59.39	60.03	59.79
**Primer reverse TM**	59.96	59.79	60.18
**Primer forward GC %**	55	55	52.4
**Primer forward GC %**	60	52.4	55
**Primer Pair Product Size (bp)**	73	94	71
**Primer Forward Genomic Span**	2L:28525774-28525852	2L:28525707-28525726	2L:28525780-28525859
**Primer Reverse Genomic Span**	2L:28525886-28525905	2L:28525780-28525859	2L:28525890-28525909

### Interrogating the ag3 resource

The malariagen_data python package pulls in Ag1000g or Af1000 data from the cloud, facilitating the rapid analysis of over 15,000 *An. gambiae s.l* or *An. funestus* whole genomes from throughout sub-Saharan Africa. In step 1 of the primer design process, we record the genomic positions of the designed primers, and in step 2 use these coordinates to extract SNP allele frequency information for given Ag1000g samples of choice. In the Colaboratory notebook, we generate a summary table of the Ag1000g inventory, counting samples by taxon, sample set and country, to guide users in selecting an appropriate cohort. Through the use of sample set identifiers, and sample queries (following standard pandas syntax), users may select any group of samples in the dataset to interrogate. Alternatively, the default settings will use every available mosquito genome. A sample query can be performed on any column of the sample metadata, such as selecting a specific species (taxon), country, year or location, amongst other metadata.

**Figure 2.**
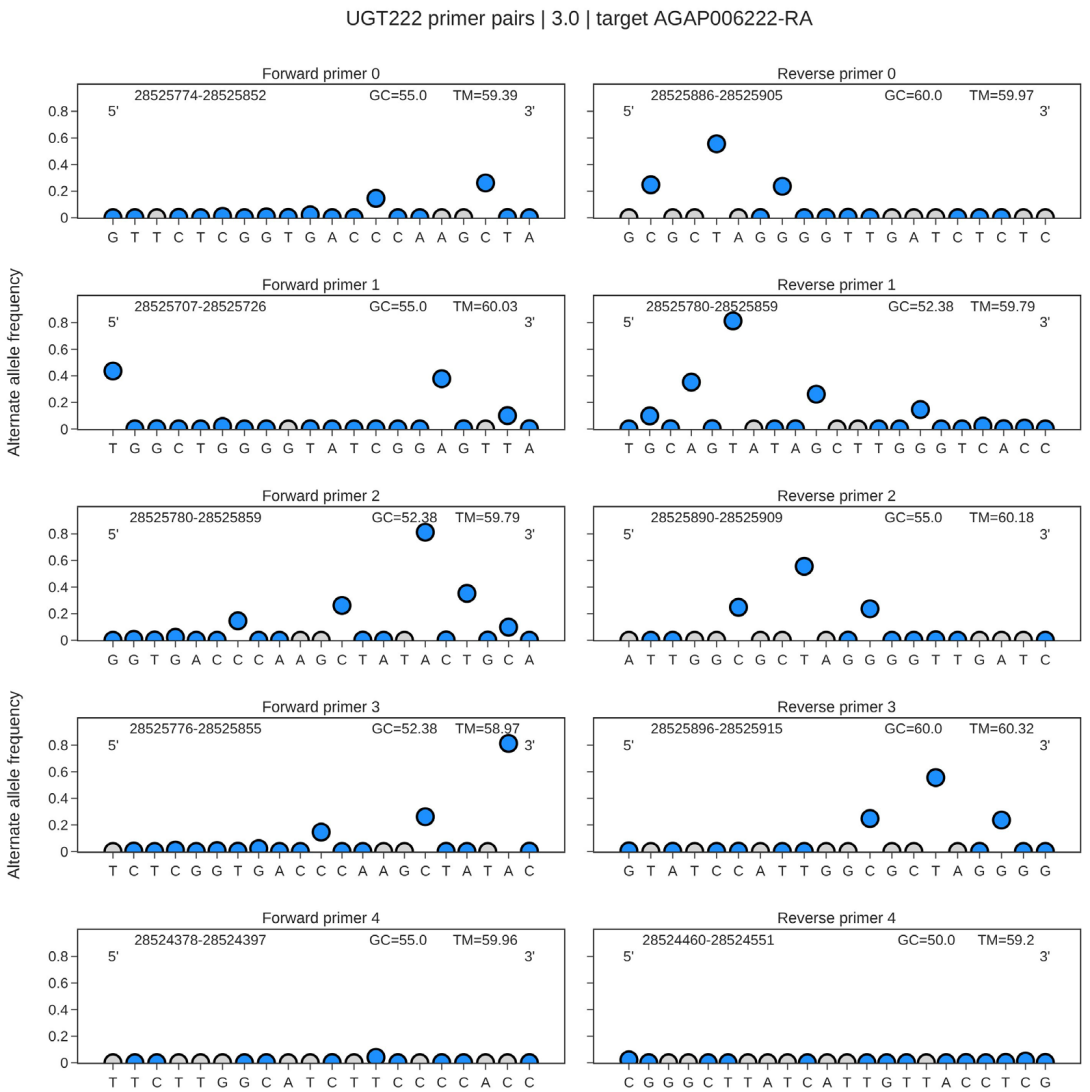
Illustrative plots showing allele frequencies in primer binding sites targeting the AGAP006222-RA transcript in specimens of *An. gambiae* and *An. coluzzii* from Ghana. An interactive plot with Plotly displays the primer or probe sequences from 5’ to 3’, with circles indicating the summed alternate allele frequency at that genomic position. Blue circles indicate segregating SNPs, and grey circles indicate sites which are invariant in the ag3 cohort of choice. The genomic span of each oligo is displayed alongside the GC content and Tm.

We then generate an interactive plot ( Figure 2) which shows SNP variation in designed primer binding sites, in the user-selected Ag1000g cohort. The user can hover over points, which returns the exact frequencies of each nucleotide at that genomic position, which may be useful in the case where the user would prefer to design degenerate primers, as opposed to avoiding that primer set entirely. The plot also highlights the 3’ and 5’ prime ends, as well as the genomic span, GC content and melting temperature, allowing the user to easily and rapidly identify suitable oligonucleotides.

### Genomic location of primers

**Figure 3.**
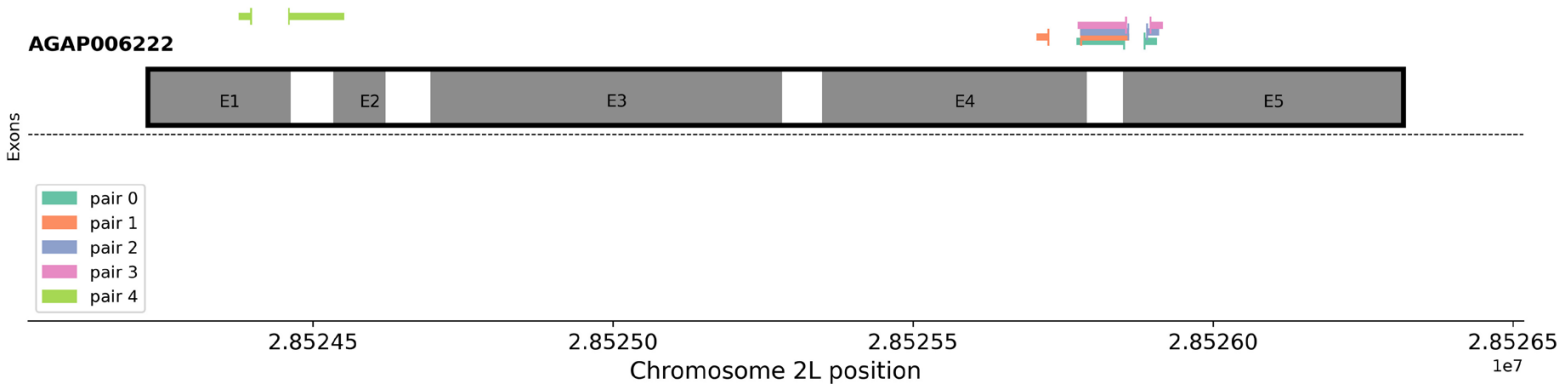
The genomic locations of designed primer sets in relation to nearby exons. Primers spanning exons are shown as expanded to clearly illustrate the span of the whole junction for visualisation purposes, and only contain sequence at each extremity.

AnoPrimer then plots the position of the primer in the genome in relation to any nearby exons. In Figure 3, we can see that all but one primer pair were designed at the Exon 4 and 5 boundary. Primer pair 4, which targets the Exon 1 and 2 junction, contains much less SNP variation than the other primers.

To ensure the specificity of the designed primer and probes for only one genomic location, we align oligonucleotides to the AgamP3 genome with BLAT, using the gget python package API ( Luebbert & Pachter, 2023).

### Testing oligos designed with AnoPrimer

To evaluate primers designed with AnoPrimer, we designed a pair of genomic DNA primers to target the *Vgsc-*V402L mutation. This mutation, alongside *Vgsc-I1527T*, is involved in resistance to pyrethroid insecticides and a haplotype containing both mutations has recently spread throughout the range of *An. coluzzii ( Clarkson *et al*., 2021; Ibrahim *et al*., 2023)*.

**Figure 4.**
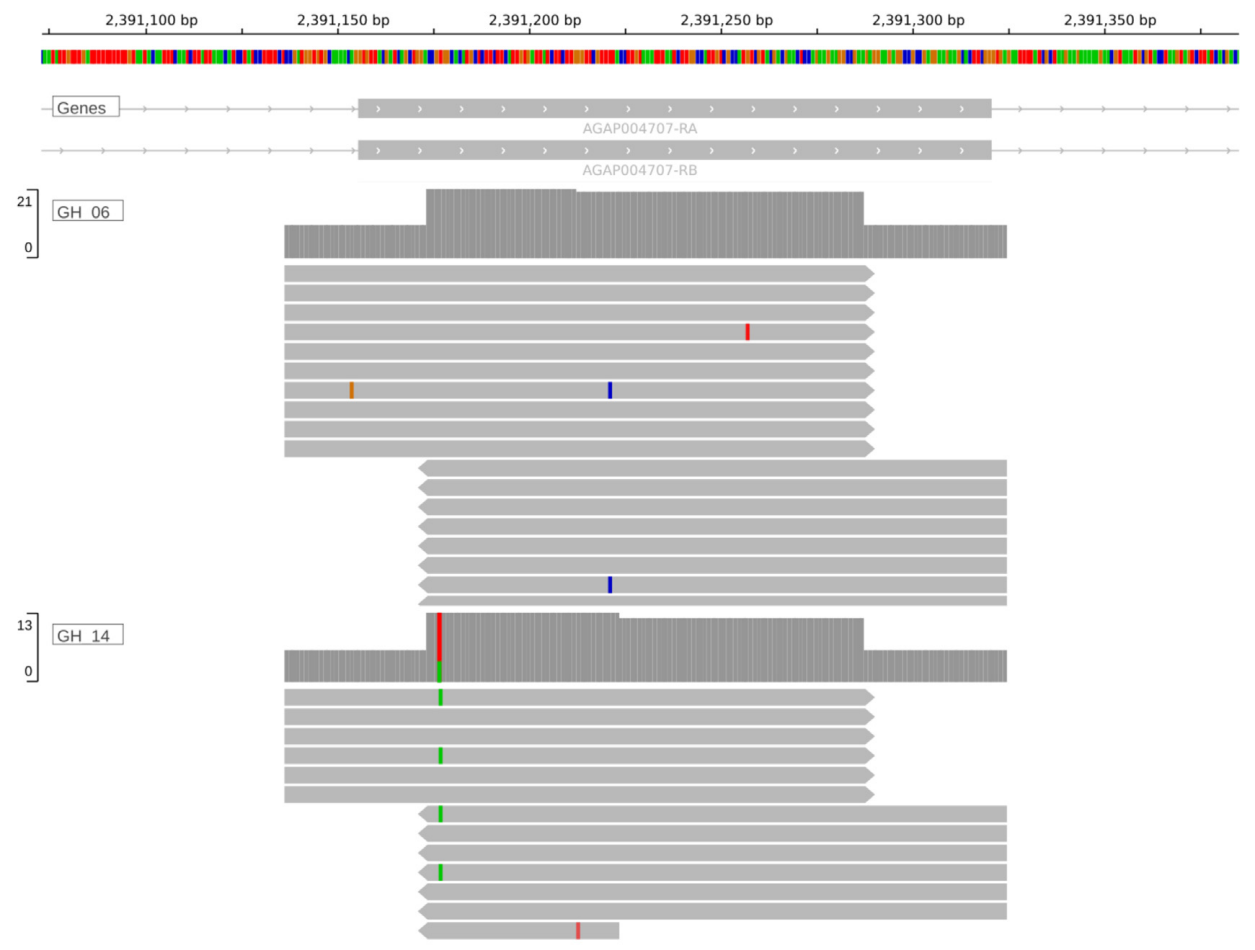
Coverage at the Vgsc-V402L locus in the integrative genomics viewer (IGV). Two representative samples of *An. gambiae* from Ghana are shown. Data is from an Illumina amplicon sequencing panel which includes primers designed by AnoPrimer targeting the Vgsc-V402L locus. Read data was loaded directly from binary alignment files (BAM).

We first used AnoPrimer to design genomic DNA primers whilst avoiding SNP variation, tested the primers in singleplex to ensure a PCR product of expected size, and then included them in a multiplex PCR as part of an amplicon sequencing panel into insecticide resistance. We used a custom library preparation protocol and ran the amplicon panel on an Illumina MiSeq instrument. Figure 4 displays the alignments in IGV at the *Vgsc-*V402L locus, demonstrating coverage at the target locus.

## Discussion

Designing reliable primers is essential in molecular biology applications, and yet, poorly designed primers can cause errors in assays which are difficult or impossible to detect. While avoiding single-nucleotide polymorphisms is commonplace in human studies, few organisms have enough robust, range-wide genetic data to do this.

AnoPrimer integrates Primer3-py and the malariagen_data API to rapidly and conveniently design variation-informed primers and probes for molecular biology. Through the use of forms in Colaboratory, users are able to define their own parameters, which means that the AnoPrimer notebook does not require programming skills. This is an extremely important point, as we hope the tool will be useful for all researchers including molecular biologists who may not have programming experience.

Genomic surveillance of malaria mosquitoes is becoming increasingly important, with a number of high throughput amplicon sequencing panels having been developed to identify species across the entire *Anopheles* genus ( Boddé *et al.*, 2022; Makunin *et al.*, 2022), within the *Anopheles gambiae* complex ( Caputo *et al.*, 2021), and to karyotype samples ( Love *et al.*, 2020). In the near future, it is likely that other amplicon sequencing panels will be designed to target phenotypes of interest, such as insecticide resistance, gene drive resistance, or vector competence. Just as in more standard genotyping assays, in amplicon sequencing robust primer design is crucial, and therefore having a computational framework to design primers is invaluable. We have demonstrated that primers designed by AnoPrimer work effectively in Illumina amplicon sequencing panels. Although AnoPrimer is capable of designing primers for use in multiplex PCR, it is designed primarily for single-locus studies, and so for this purpose, we recommend the tool Multiply ( de Cesare *et al.*, 2024).

## Ethics and consent

Ethical approval and consent were not required.

## Data Availability

All data used in this project is openly accessible through the malariagen_data API. The data in Table 1, and Figure 2 and Figure 3 are generated using the AnoPrimer.designPrimers() function, using the following parameters; sample_sets: ‘AG1000G-GH’, target: ‘AGAP006222-RA’, assay_type: ‘cDNA_primers’, species: ‘gambiae_sl’, n_primer_pairs: 5, min_amplicon_size: 60, max_amplicon_size: 120.

## Software availability

AnoPrimer is open-source and free to use, and we encourage suggestions and contributions to the software. The package is available here https://github.com/sanjaynagi/AnoPrimer and on the Python package index (PyPi). Archived source code can be accessed through Zenodo ( https://zenodo.org/doi/10.5281/zenodo.6913822 ( Nagi & Miles, 2024)). The software is licensed under an MIT license.
