# Supplementary material for: AnoPrimer: Primer Design in malaria vectors informed by range-wide genomic variation
Source: Wellcome Open Res. 2024 May 15;9:255. [Version 1] doi: 10.12688/wellcomeopenres.20998.1 (PMC11342028; doi:10.12688/wellcomeopenres.20998.1)
Supplement: Peer review history

# Reviewer report: Reviewer response for version 1

**DOI:** 10.21956/wellcomeopenres.23233.r91093

**Published:** 2024-08-22

**Authors:** Supriya Sharma

**Affiliations:** 1 ICMR-National Institute of Malaria Research, New Delhi, India

This article presents a new software tool, AnoPrimer, for designing a robust primer for PCR assays in highly genetically variable organisms—the major malaria mosquito vectors *Anopheles gambiae* and *An. funestus*. Traditional primer design methods ignore existing genetic variation, which may incorporate potential errors into downstream applications. AnoPrimer integrates open-source tools  to harness the power of cloud-based genomics data from *Anopheles* 1000 Genomes projects in primer design that would avoid known SNPs. These conclusions are thus partly supported by the findings but could be more broadly validated. This paper gives evidence that AnoPrimer is effective only in one use case. Ideally, the authors would give additional examples targeting other genes and functionalities, such as multiplex PCR primer design (though they mention some limitations of AnoPrimer for this particular task).

**Improvement Key Points:**

- More details on the implementation of Primer3-py and malariagen_data functionalities within AnoPrimer.
- Supplement with source code repositories or pseudocode examples for the sake of ease in replication.
- Expand AnoPrimer validation by showing its efficiency for primer design for many genes and various applications beyond single-locus studies.

By doing so, the authors can make the paper more technically sound and simplify using AnoPrimer for the research community.

 Using a use case example to describe AnoPrimer functionality, they have targeted the *Vgsc*-V402L mutation, a mutation conferring pyrethroid insecticide resistance in *An. coluzzii*, where they designed primers. Using these designed primers, amplicon sequencing was performed, which successfully amplified the target locus.

Are the conclusions about the tool and its performance adequately supported by the findings presented in the article?

Yes

Is the rationale for developing the new software tool clearly explained?

Yes

Is the description of the software tool technically sound?

Yes

Are sufficient details of the code, methods and analysis (if applicable) provided to allow replication of the software development and its use by others?

Yes

Is sufficient information provided to allow interpretation of the expected output datasets and any results generated using the tool?

Yes

Reviewer Expertise:

Malaria molecular research

I confirm that I have read this submission and believe that I have an appropriate level of expertise to confirm that it is of an acceptable scientific standard.

# Reviewer report: Reviewer response for version 1

**DOI:** 10.21956/wellcomeopenres.23233.r91089

**Published:** 2024-08-22

**Authors:** Katlijn De Meulenaere

**Affiliations:** 1 Institute of Tropical Medicine Antwerp, Antwerp, Belgium

Genetic diversity is very high in *Anopheles* species, which makes primer design complex. AnoPrimer takes into account genetic variation collected by MalariaGEN to aid primer design, while also taking advantage of the widely used Primer3 tool for primer design. The documentation is clear, the tool is easily accessible for non-bioinformaticians in two different google Colab notebooks, while a python package is available as well, and all code is made publicly available. Furthermore, it is helpful that users can select specific taxa, years or countries within the MalariaGEN dataset to take the relevant SNPs into account for their primer design. Taken together, this promises to be a valuable tool for the *Anopheles* community.

**Minor comments or suggestions:**

- In the google Colab notebook, users will run into a permission error when they do not yet have access to the relevant MalariaGEN datasets. This is explained in the Documentation, but it would be helpful if a link to the MalariaGEN data access form ( MalariaGEN cloud data access form) is directly added above the relevant cell in the Google Colab notebook.
- In the paper, it is written that "To ensure the specificity of the designed primer and probes for only one genomic location, we align oligonucleotides to the AgamP3 genome with BLAT". Does this step also exist for *Anopheles funestus*?
- In the long version of the Google Colab notebook, I missed the option of choosing a taxon or country at the start ( “sample_query”).
- At the very end of the discussion, it is said that AnoPrimer can be used to design Illumina amplicon sequencing panels, after which it is recommended to do this with another tool, Multiply. How does AnoPrimer3 compare to Multiply in sense of SNP detection? Is Multiply always preferred when you multiplex, or is AnoPrimer3 better for SNP design, or would you combine both tools for an optimal result?
- Will this tool support new versions of the MalariaGEN datasets?

Are the conclusions about the tool and its performance adequately supported by the findings presented in the article?

Yes

Is the rationale for developing the new software tool clearly explained?

Yes

Is the description of the software tool technically sound?

Yes

Are sufficient details of the code, methods and analysis (if applicable) provided to allow replication of the software development and its use by others?

Yes

Is sufficient information provided to allow interpretation of the expected output datasets and any results generated using the tool?

Yes

Reviewer Expertise:

Bioinformatics (malaria blood stages)

I confirm that I have read this submission and believe that I have an appropriate level of expertise to confirm that it is of an acceptable scientific standard.

# Reviewer report: Reviewer response for version 1

**DOI:** 10.21956/wellcomeopenres.23233.r91091

**Published:** 2024-08-22

**Authors:** Melina Campos

**Affiliations:** 1 UC Davis, Davis, CA, USA

**Review of "AnoPrimer: A Python Package for Designing Reliable Primers and Probes for** ***Anopheles gambiae*** **and** ***Anopheles funestus*** **"**

 The paper by Nagi et al. introduces AnoPrimer, a Python package for designing primers tailored to two malaria vectors in Africa, *Anopheles gambiae* and *Anopheles funestus*. This tool leverages genetic variants identified in field-collected populations across Africa to optimize primer design, thereby potentially enhancing the performance of amplification assays. AnoPrimer utilizes two whole genome sequencing databases: Ag1000G and Af1000.

**After reviewing the paper, I have two suggestions that could enhance the tool's usability and accessibility to a broader audience:**

- The specific codes used for the databases, such as AG1000G-GH, should be made easily accessible and clearly explained, as their meaning may not be immediately understood by those unfamiliar with the database.
- While the authors state that users do not require programming skills, the reliance on Python could still present a barrier for those lacking the technical expertise. Developing a web interface would help overcome this issue and make the tool more accessible to a broader audience.

Are the conclusions about the tool and its performance adequately supported by the findings presented in the article?

Yes

Is the rationale for developing the new software tool clearly explained?

Yes

Is the description of the software tool technically sound?

Yes

Are sufficient details of the code, methods and analysis (if applicable) provided to allow replication of the software development and its use by others?

Yes

Is sufficient information provided to allow interpretation of the expected output datasets and any results generated using the tool?

Yes

Reviewer Expertise:

population genetics, genomics, bioinformatics

I confirm that I have read this submission and believe that I have an appropriate level of expertise to confirm that it is of an acceptable scientific standard.

# Reviewer report: Reviewer response for version 1

**DOI:** 10.21956/wellcomeopenres.23233.r91092

**Published:** 2024-08-06

**Authors:** Konstantinos Mavridis

**Affiliations:** 1 Institute of Molecular Biology and Biotechnology, Foundation for Research and Technology-Hellas, Heraklion, Greece

The paper "AnoPrimer: A Python Package for Designing Reliable Primers and Probes for Anopheles gambiae and Anopheles funestus" presents a well-constructed Python package that makes use of the Ag1000G and Af1000 datasets to aid in the design of primers and probes for these genetically diverse mosquito species. The tool is designed to account for genetic variation in primer- and most importantly probe- binding sites, which is crucial for avoiding amplification issues such as null alleles or preferential amplification. The primary limitation of AnoPrimer is its reliance on Python, which can be a barrier for researchers who are not familiar with programming. This could reduce its accessibility to the broader molecular biology community. 

 To enhance the usability and accessibility of AnoPrimer, the following  are proposed:

- Develop a user-friendly web interface that allows researchers to use AnoPrimer. This interface could include forms to input sequence data, options to select primer design parameters, and buttons to execute the analysis and ensure the web interface provides real-time feedback and visualizations, similar to the current Python package.

Are the conclusions about the tool and its performance adequately supported by the findings presented in the article?

Yes

Is the rationale for developing the new software tool clearly explained?

Yes

Is the description of the software tool technically sound?

Yes

Are sufficient details of the code, methods and analysis (if applicable) provided to allow replication of the software development and its use by others?

Yes

Is sufficient information provided to allow interpretation of the expected output datasets and any results generated using the tool?

Yes

Reviewer Expertise:

Molecular diagnostics.

I confirm that I have read this submission and believe that I have an appropriate level of expertise to confirm that it is of an acceptable scientific standard.
